# Asociación entre parámetros hemáticos y el desarrollo de nefropatía inducida por contraste en pacientes con infarto de miocardio sin elevación del segmento ST

**DOI:** 10.1515/almed-2023-0119

**Published:** 2023-09-21

**Authors:** Esra Dönmez, Sevgi Özcan, İrfan Şahin, Ertuğrul Okuyan

**Affiliations:** Departamento de Cardiología, Bağcılar Training and Research Hospital, Bağcılar, İstanbul, Turquía

**Keywords:** nefropatía inducida por contraste, NIC, volumen plaquetario medio, índice neutrófilo/linfocito

## Abstract

**Objetivos:**

Los parámetros hemáticos, como el volumen plaquetario medio (VPM), el índice neutrofilo/linfocito (INL), la amplitud de distribución eritrocitaria (ADE), y la amplitud de distribución plaquetaria (ADP), son indicadores ampliamente utilizados en el pronóstico de diversas patologías cardiovasculares. Investigamos el valor predictivo de los parámetros hemáticos en el desarrollo de nefropatía inducida por contraste (NIC), en pacientes con infarto de miocardio sin elevación del segmento ST (IAMSEST), sometidos a intervención coronaria percutánea (ICP).

**Métodos:**

Se incluyó retrospectivamente a todos los pacientes diagnosticados de IAMSEST que se sometieron a una ICP percutánea entre 2017 y 2020 en un hospital terciario.

**Resultados:**

Se incluyó a un total de 387 pacientes. El análisis de regresión logística mostró que la edad avanzada (p=0,001, β:0,005, OR [IC95 %]: 0,002–0,007), la presencia de diabetes mellitus (p=0,013, β:0,205, OR [IC95 %]: 0,150–0,260), insuficiencia cardíaca congestiva (p=0,009, β:0,095, OR [IC95 %]: 0,024–0,166), así como el volumen plaquetario medio (p=0,008, β:0,241, OR [IC95 %]: 0,184–0,392), VPM (p=0,02, β:0,047, OR [IC95 %]: 0,028–0,065), e INL (p=0,001, β:0,052, OR [IC95 %]: 0,040–0,063) fueron factores de riesgo independientes de desarrollar NIC. Un valor umbral de 5,5 para INL mostró una sensibilidad del 79,6 % y una especifidad del 79,5 %, mientras que el valor umbral de 9,05 para el VPM mostró una sensibilidad del 64,1 % y una especifidad del 58,7 % a la hora de predecir el desarrollo de NIC.

**Conclusiones:**

los parámetros hematológicos, medidos mediante hemograma rutinario, se postulan como marcadores útiles y prometedores de NIC, cuando se emplean en combinación con otros factores de riesgo habituales. El VPM y el INL predicen el desarrollo de NIC en pacientes con IAMSEST sometidos a ICP.

## Introducción

La nefropatía inducida por contraste (NIC) es una tipología de daño renal agudo asociado al empleo de un medio de contraste (MC) intravascular, así como una de las complicaciones más conocidas de los procedimientos de cateterismo cardíaco. El NIC se define como la elevación de los niveles de creatinina en suero (Cr) en al menos 0,5 mg/dL o del 25 % con respecto a la Cr basal en suero, transcurridas entre 48 y 72 horas desde la exposición al medio de contraste [1]. Su incidencia en la población general es de entre el 2 y el 5 %, aproximadamente. Sin embargo, dicha prevalencia puede incrementarse ante la presencia de algunos factores relacionados con el paciente [comorbilidades como una edad avanzada, enfermedad renal crónica (ERC), diabetes mellitus (DM), insuficiencia cardíaca congestiva (ICC)], así como con el procedimiento (síndrome coronario agudo, intervenciones coronarias complejas) y la cantidad de MC (hasta un 20–30 %) [2]. Dado que el desarrollo de la NIC está asociado a una mayor morbimortalidad intrahospitalaria y a largo plazo, el diagnóstico temprano, así como la identificación de aquellos pacientes con mayor riesgo, podrían mejorar la progresión y el pronóstico de la enfermedad [3, 4].

La vasoconstricción, la toxicidad celular directa y la inflamación son factores de riesgo de desarrollar NIC [5], [6], [7]. Los parámetros hemáticos, como el volumen plaquetario medio (VPM), el índice neutrofilo/linfocito (INL), la amplitud de distribución eritrocitaria (ADE), y la amplitud de distribución plaquetaria (ADP) son indicadores ampliamente utilizados en el pronóstico de diversas patologías cardiovasculares [8], [9], [10], [11]. La evidencia sobre el papel de los parámetros hemáticos en el desarrollo de la NIC es limitada. En el presente estudio, se investigó el papel de los parámetros hemáticos a la hora de predecir el desarrollo de nefropatía inducida por contraste (NIC) en pacientes con infarto de miocardio sin elevación del segmento ST (IAMSEST), sometidos a intervención coronaria percutánea (ICP).

## Materiales y métodos

Se incluyó retrospectivamente a todos los pacientes con diagnóstico de IAMSEST, sometidos a una ICP entre 2017 y 2020 en un hospital terciario. Se extrajeron datos demográficos, clínicos y analíticos de las bases de datos del hospital, así como del historial médico de los pacientes. Así mismo, se registraron los tratamientos médicos administrados en el hospital, así como previamente a la hospitalización. El diagnóstico de infarto de miocardio sin elevación del segmento ST se estableció conforme a las guías clínicas más recientes [12]. La enfermedad multivaso se definió como la presencia de una estenosis ≥50 % en dos o más arterias coronarias epicárdicas Se excluyó a aquellos pacientes con leucemia, trombocitopenia, enfermedad renal terminal, cáncer, enfermedad autoinmune crónica y/o pacientes bajo terapia antiinflamatoria con esteroides o no esteroides y exposición a medios de contraste en las últimas dos semanas. También se excluyó a los pacientes a los que se administró tratamiento médico, ICP multivaso o cirugía emergente basada en angiografía coronaria. En nuestro hospital, como tratamiento de seguimiento rutinario, se administra solución salina (0,9 %) por vía intravenosa a una velocidad de 1 mL/kg/h y 0,5 mL/kg/h a los pacientes con una fracción de eyección reducida (FE) o ICC, en las 12 h posteriores a la exposición al contraste. En nuestro laboratorio de cateterismo, se utilizó un contraste no iónico de baja osmolalidad.

El diagnóstico de insuficiencia cardíaca congestiva se basó en antecedentes de insuficiencia cardíaca previa o evidencia objetiva de una fracción de eyección del ventrículo izquierdo reducida (FEVI) ≤40 %, evaluada mediante electrocardiograma en el momento del ingreso [13]. Todos los pacientes se sometieron a un ecocardiograma transtorácico (Vivid S70; GE Medical System, Horten, Noruega), mientras que la fracción de eyección ventricular izquierda (FEVI) se midió con el método de Simpson. La hipertensión (HT) se confirmó con la prescripción de tratamientos farmacológicos para reducir la presión arterial, cualquier resultado superior a 140/90 mmHg previo a la operación y/o un diagnóstico oficial previo [14]. El accidente cerebrovascular, o ictus, se definió como todo antecendente de focalidad neurológica de una duración superior a 24 horas, producto de una alteración en el flujo sanguíneo del cerebro [15]. La disfunción neurológica reversible que provocara síntomas que persistieran durante menos de 24 horas se definió como accidente isquémico transitorio (TIA) [16]. Un nivel de glucosa en ayunas de ≥126 mg/dL (7,0 mmol/L) y/o un valor de hemoglobina A_1c_ >6,5 %, o la toma de fármacos para la diabetes eran indicativos de la presencia de diabetes mellitus (DM) [17]. La arteriopatía coronaria (AC) se definió como una estenosis del 50 % del diámetro luminal en al menos un vaso epicárdico mayor, observada mediante angiografía coronaria diagnóstica.

Se tuvieron en cuenta los parámetros hemáticos (hemoglobina, ADE, PDE, VPM, recuento plaquetario, de neutrofilos, monocitos y eosinófilos) obtenidos en el primer ingreso del que se tuviera constancia (Cell Dyn 3700; Abbott Diagnostics, Lake Forest, Illinois, EE.UU). “Cr basal” se definió como el nivel de Cr basal en suero obtenido mediante análisis bioquímico en el primer ingreso. El “valor máximo de Cr” se estableció como el nivel de Cr en suero obtenido, transcurridas al menos 48 horas desde la administración del contraste. Se dividió a los pacientes en dos grupos, según si desarrollaron o no NIC. Así, el grupo de pacientes que desarrollaron NIC se denominó NIC (+), mientras que el grupo de pacientes que no lo desarrollaron recibió el nombre de CIN (−). El criterio de valoración principal de este estudio fue la aparición de NIC. El Comité de Investigacion en Humanos de nuestra institución aprobó el estudio, por lo que no fue necesario solicitar el consentimiento de los pacientes.

### Análisis estadístico

Todos los análisis estadísticos se realizaron empleando el programa Statistical Package for the Social Sciences 22.0 (SPSS Inc., Chicago, IL, EE.UU). Las variables continuas se expresan como medias ± DE, mientras que los datos categóricos se presentan en valores absolutos (n) y porcentuales (%). Para investigar diferencias en las variables categóricas entre los grupos, se utilizó la prueba de Chi cuadrado. La prueba t de Student o la prueba U de Mann Whitney se emplearon para comparar las muestras no pareadas, en su caso. Las variables que mostraron una correlación lineal se evaluaron mediante la prueba de correlación de Pearson, mientras que las variables no lineales se evaluaron mediante la prueba de correlación de Spearman. Se investigó la posible correlación entre los parámetros hemáticos y el desarrollo de NIC. Empleamos el análisis de regresión logística para identificar variables independientes relacionadas con el desarrollo de NIC en la población de estudio. Se calculó el área bajo la curva ROC para evaluar la capacidad de los parámetros hemáticos para predecir los resultados clínicos. Un p bilateral <0,05 se consideró estadísticamente significativo.

## Resultados

Se incluyó a un total de 529 pacientes, una vez excluidos los pacientes que cumplían los criterios de exclusión descritos en la metodología, así como aquellos cuyos datos estaban incompletos. La muestra final estuvo compuesta por 387 pacientes. La edad media fue de 65,9 ± 10,9, y el 60,5 % eran hombres. El valor medio de Cr basal fue de 1,05 ± 0,28. Los pacientes fueron agrupados según si desarrollaron NIC (+) o no (−). Un total de 74 (19,1 %) pacientes fueron incluidos en el grupo NIC (+), mientras que 313 (80,9 %) se incluyeron en el grupo NIC (−). Ambos grupos eran similares en relación al género, índice de masa corporal, hábito tabáquico, hiperlipidemia, HT, antecedentes de accidente cerebrovascular y tratamiento médico recibido. Sin embargo, observamos diferencias estadísticamente significativas, ya que los pacientes del grupo NIC (+), tenían una edad más avanzada (70,4 ± 9,2 vs. 64,8 ± 11,1; p<0,0001), y mayor incidencia de DM (66,2 % vs. 26,2 %; p<0,0001), arteriopatía coronaria (AC) (64,8 % vs. 32,3 %; p<0,0001), e ICC (40,5 % vs. 10,5 %; p<0,0001), y mayor volumen de MC administrado (142 ± 39 vs. 101 ± 34; p=0,004). En cuanto a los marcadores analíticos, la urea máxima (78,2 ± 46,4 vs. 46,7 ± 19,3; p<0,0001), la Cr máxima (1,69 ± 0,74 vs. 1,04 ± 0,29; p<0,0001), ácido úrico (7,1 ± 1,9 vs. 5,9 ± 2,2; p=0,003), troponina [40,6 (4,3–5000) vs. 32,0 (1–3590); p=0,002], y la glucemia en ayunas (169,9 ± 85,4 vs. 140,1 ± 70,2; p=0,002) fueron significativamente mayores, mientras que los niveles de albúmina (3,8 ± 0,5 vs. 4,2 ± 0,7; p=0,002) fueron significativamente inferiores en el grupo NIC (+). Además, el ADP (16,2 ± 4,3 vs. 14,8 ± 2,7; p=0,016), VPM (9,4 ± 2,5 vs. 8,2 ± 1,3; p=0,035), y el INL (5,3 ± 1,8 vs. 3,9 ± 1,1; p<0,0001) fueron significativamente superiores en el grupo NIC (+). En las Tablas 1 y 2 se detallan las características demográficas, clínicas y analíticas de los dos grupos.

**Tabla 1:** Datos clínicos y demográficos de la población de estudio y dos grupos según el desarrollo o no de nefropatía.

Variables	Todos	Grupo-1	Grupo-2	Valor p
		CIN (+)	NIC (−)	
	n=387	n=74	n=313	
**Características clínicas y comorbilidades**				

Edad, años	65,9 ± 10,9	70,4 ± 9,2	64,8 ± 11,1	**<0,0001**
Hombres, n (%)	234 (60,5)	44 (59,5)	190 (60,7)	0,844
IMC, kg/m^2^	28,7 ± 4,4	28,5 ± 4,4	28,7 ± 4,4	0,673
Fumador, n (%)	102 (26,5)	18 (24,3)	84 (27,0)	0,638
HT, n (%)	224 (57,9)	45 (60,8)	179 (57,2)	0,068
HPL, n (%)	42 (10,9)	9 (12,2)	33 (10,5)	0,687
DM, n (%)	131 (33,9)	49 (66,2)	82 (26,2)	**<0,0001**
Antecendente de AC, n (%)	149 (38,5)	48 (64,8)	101 (32,3)	**<0,0001**
Ictus previo, n (%)	11 (2,8)	2 (2,7)	9 (2,9)	0,586
ICC previa, n (%)	63 (16,3)	30 (40,5)	33 (10,5)	**<0,0001**
FEVI, %	48,2 ± 11,2	46,4 ± 11,6	48,6 ± 11,1	0,176
Volumen de MC, mL	119 ± 62	142 ± 39	101 ± 34	**0,004**
Fármacos, n (%)				
Aspirina	292 (75,4)	58 (78,4)	234 (74,8)	0,136
Inhibidor de ECA/uso de ARA	219 (56,5)	44 (59,5)	175 (55,9)	0,084
Bloqueador del canal de calcio	101 (26,1)	17 (22,9)	84 (26,8)	0,097
β-bloqueante	127 (32,8)	24 (32,4)	103 (32,9)	0,584

**Parámetros analíticos**				

Urea, mg/dL				
Basal	45,1 ± 19,5	47,7 ± 19,6	44,5 ± 19,4	0,200
Transcurridas 48 h	52,6 ± 29,3	78,2 ± 46,4	46,7 ± 19,3	**<0,0001**
Creatinina, mg/dL				
Basal	1,05 ± 0,28	1,11 ± 0,29	1,03 ± 0,28	0,059
Transcurridas 48 h	1,16 ± 0,49	1,69 ± 0,74	1,04 ± 0,29	**<0,0001**
Hemoglobina, g/L	13,2 ± 1,8	12,8 ± 1,9	13,1 ± 1,9	0,385
Hematocrito, %	39,4 ± 5,3	39,1 ± 5,3	39,4 ± 5,2	0,691
ADP, %	15,7 ± 3,9	16,2 ± 4,3	14,8 ± 2,7	**0,016**
ADE, %	14,1 ± 1,5	13,9 ± 1,5	14,1 ± 1,5	0,705
VPM, fL	8,7 ± 1,4	9,4 ± 2,5	8,2 ± 1,3	**0,035**
WBC×10³/μL	8,47 ± 4,92	8,67 ± 5,76	7,89 ± 4,39	0,087
Recuento plaquetario 10³/μL	223 (147–494)	219 (147–481)	217 (149–494)	0,258
INL	3,7 ± 2,1	5,3 ± 1,8	3,9 ± 1,1	**<0,0001**
Albúmina, g/dL	4,3 ± 0,6	3,8 ± 0,5	4,2 ± 0,7	**0,002**
Ácido úrico, mg/dL	6,2 ± 2,2	7,1 ± 1,9	5,9 ± 2,2	**0,003**
ProBNP, pg/mL	77,8 (50–28679)	96,5 (186–28679)	76,1 (50–26528)	0,439
Troponina T, pg/mL	39,0 (1–5000)	40,6 (4,3–5000)	32,0 (1–3590)	**0,002**
Glucosa en ayunas, mg/dL	145,9 ± 74,2	169,9 ± 85,4	140,1 ± 70,2	**0,002**
AST, U/L	26,0 (8–1,245)	31,0 (8–1,245)	25,0 (10–1,105)	0,079
ALT, U/L	23,0 (5–540)	21,0 (9–540)	25,0 (5–540)	0,101
Colesterol total, mg/dL	184,1 ± 52,2	182,6 ± 43,3	184,5 ± 52,9	0,782
Triglicéridos, mg/dL	170,9 ± 112.5	147,4 ± 57,2	176,4 ± 121,3	0,052
Colesterol LDL, mg/dL	111,3 ± 39,3	111,9 ± 35,4	111,2 ± 40,2	0,878
Colesterol HDL, mg/dL	41,7 ± 11,9	41,5 ± 9,4	41,8 ± 12,5	0,873

ECA/ARA, inhibidor de la enzima convertidora de angiotensina/antagonista de los receptores de la angiotensina; ALT, alanina aminotransferasa; AST, aspartato aminotransferasa; IMC, índice de masa corporal; PNC, péptido natriurético cerebral; AC, arteriopatía coronaria; ICC, insuficiencia cardíaca congestiva; NIC, neuropatía inducida por contraste; MC, medio de contraste; DM, diabetes mellitus; HDL, lipoproteína de alta densidad; HPL, hiperlipidemia; HT, hipertensión; LDL, lipoproteína de alta densidad; FEVI, fracción de eyección del ventrículo izquierdo; VPM, volumen plaquetario medio; INL, índice neutrófilo/linfocito; PAD, arteriopatía periférica; ADP, amplitud de distribución plaquetaria; ADE, amplitud de distribución eritrocitaria; WBC, leucocitos. p<0,05 se aceptó como estadísticamente significativo.

**Tabla 2:** Análisis de regresión logística gradual univariante y multivariante: factores predictivos de NIC.

	OR univariante	IC95 %	Valor p	OR multivariante	IC95 %	Valor p
Edad, años	0,009	0,006–0,012	**<0,0001**	0,005	0,002–0,007	**0,001**
Diabetes mellitus	0,330	0,270–0,391	**<0,0001**	0,205	0,150–0,260	**0.013**
Antecedente de AC	0,339	0,343–1,456	0,127			
Antecendente de ICC	0,420	0,345–0,494	**0,001**	0,095	0,024–0,166	**0,009**
Volumen de MC	0,268	0,093–0,444	**0,003**	0,241	0,184–0,392	**0,008**
Albúmina	0,122	0,049–0,049	**0,030**	0,098	0,064–1,045	0,726
Ácido úrico	0,175	0,005–1,035	0,138			
Troponina T	0,174	0,008–1,158	0,881			
Glucosa en ayunas	0,251	0,102–1,481	0,958			
ADP	0,259	0,114–0,389	**0,020**	0,814	0,189–1,002	0,113
VPM	0,067	0,046–0,088	**0,002**	0,047	0,028–0,065	**0,020**
INL	0,050	0,034–0,067	**<0,0001**	0,052	0,040–0,063	**0,001**

AC, arteriopatía coronaria; ICC, insuficiencia cardíaca congestiva; NIC, neuropatía inducida por contraste; MC, medio de contraste; DM, volumen plaquetario medio; INL, índice neutrófilo/linfocito; PAD, amplitud de distribución plaquetaria. p<0,05 se aceptó como estadísticamente significativo.

**Figura 1:**
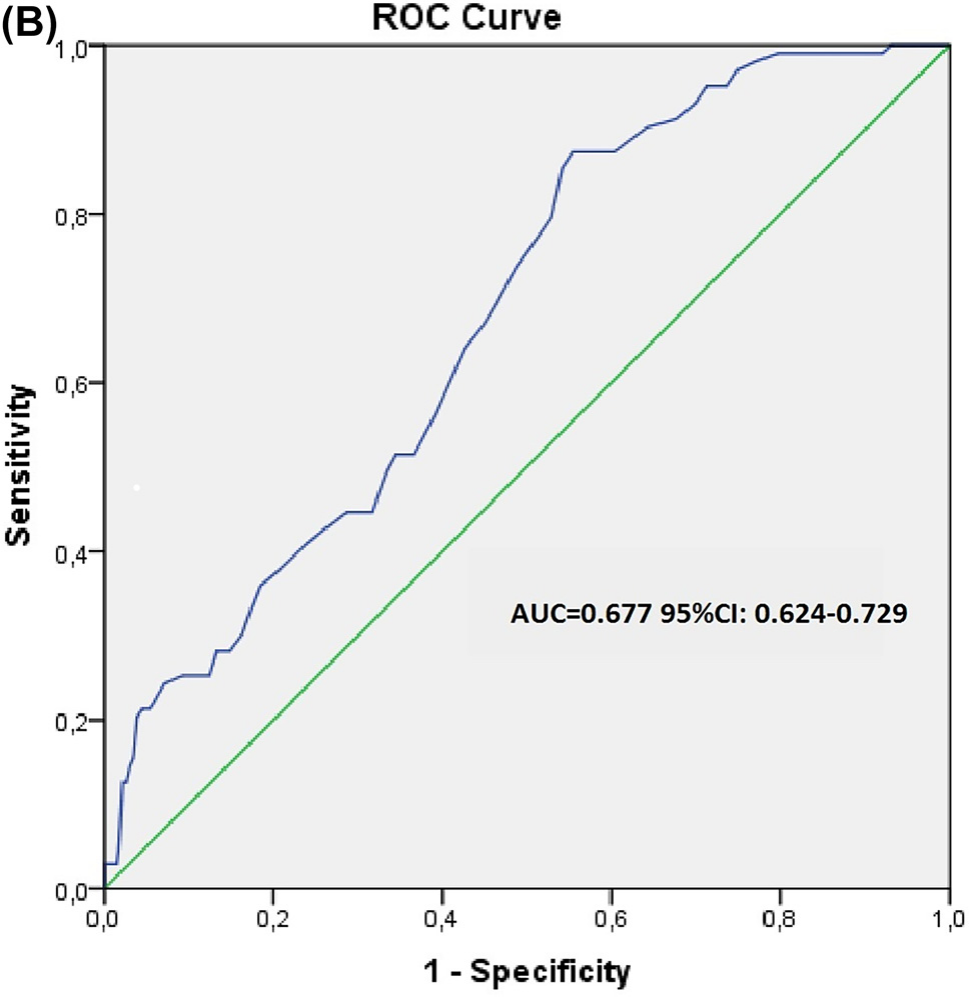
Curva ROC de la exactitud de los parámetros del hemograma para predecir el desarrollo de NIC en pacientes con IAMSEST. (A) Área bajo la curva ROC del índice neutrófilo/linfocito y desarrollo de NIC en pacientes con IAMSEST. (B) Área bajo la curva ROC de volumen plaquetario medio y desarrollo de NIC en pacientes con IAMSEST.

Para analizar con mayor profundidad los factores de riesgo individual de desarrollar NIC, se realizó un análisis de regresión logística univariante, en el que se incluyó la edad, DM, antecedentes de AC, e ICC, volumen de MC, y los niveles de albúmina, ácido úrico, troponina, glucosa en ayunas, ADP, VPM, e INL, respectivamente. El análisis de regresión logística univariante confirmó la correlación entre la edad avanzada, el volumen de MC administrado, antecedentes de DM o ICC, los niveles de albúmina, ADP, VPM e INL y el desarrollo de NIC. Dichas variables se evaluaron en el modelo de regresión logística multivariante. Según el análisis de regresión logística multivariante, la edad avanzada [p=0,001, β: 0,005, OR (IC95 %): 0,002–0,007], los antecedentes de DM [p=0,013, β: 0,205, OR (CI95 %): 0,150–0,260], ICC [p=0,009, β: 0,095, OR (IC95 %): 0,024–0,166], volumen de MC [p=0,008, β: 0,241, OR (IC95 %): 0,184–0,392], VPM [p=0,02, β: 0,047, OR (IC95 %): 0,028–0,065], e INL [p=0,001, β: 0,052, OR (CI95 %): 0,040–0,063] fueron factores de riesgo independientes de NIC. Se realizó un análisis de la curva ROC para identificar el valor umbral óptimo y el área bajo la curva (AUC) correspondiente al INL. En la Figura 1A se muestra la curva ROC de precisión del INL a la hora de predecir el desarrollo de NIC en pacientes con IAMSEST. El AUC del INL fue de 0,836 [IC95 %: 0,795–0,877]. Un valor umbral de 5,5 para INL se asoció con una sensibilidad del 79,6 % y una especifidad del 79,5 % a la hora de predecir el desarrollo de NIC. Así mismo, en la Figura 1B se muestra la curva ROC del INL a la hora de predecir el desarrollo de NIC en pacientes con IAMSEST. El AUC para el INL fue de 0,677 [IC95 %: 0,624–0,729]. Un valor umbral de 9,05 para INL se asoció con una sensibilidad del 64,1 % y una especifidad del 58,7 %, a la hora de predecir el desarrollo de NIC.

**Figure:**
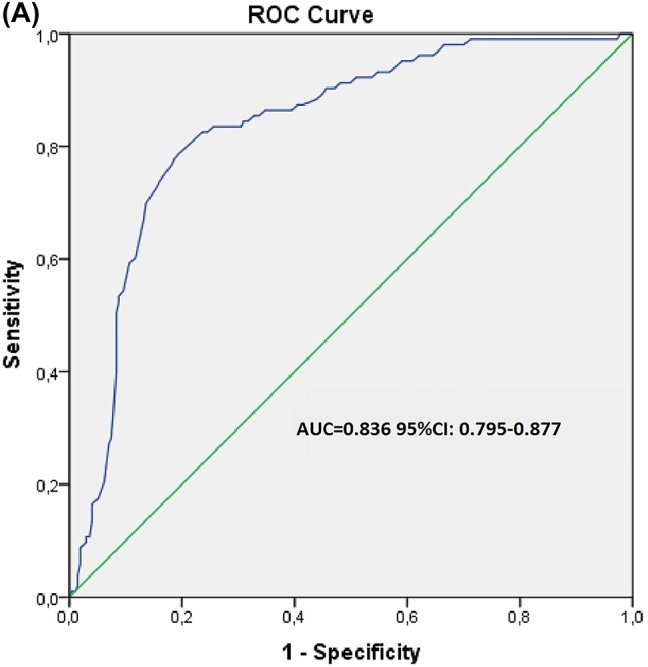


## Discusión

La nefropatía inducida por contraste tuvo una prevalencia del 19,1 %, y los parámetros hemáticos predijeron el desarrollo de NIC. El análisis preoperatorio de los parámetros hemáticos puede ayudar a predecir el desarrollo de NIC en pacientes con IAMSEST. Una edad avanzada, tener antecedentes de DM o ICC, el volumen de MC, el valor de VPM y el INL fueron factores predictivos de NIC. Un valor VPM >9,05 y un INL >5.5 en el momento del ingreso se pueden utilizar como indicadores de riesgo de NIC, si es emplean en combinación con otros factores de riesgo.

La administración de medios de contraste forma parte habitual de las técnicas diagnósticas y terapéuticas, especialmente en el campo de la cardiología, habiendo aumentado su uso, debido a los avances en el campo de la cardiología intervencionista. NIC es una complicación grave de la exposición a MC. Además de estar asociada a una mayor morbimortalidad, una estancia más prolongada en el hospital y en la unidad de cuidados intensivos, y una mayor necesidad de hemodiálisis, el desarrollo de NIC también supone un incremento de costes [18]. La inflamación, la trombosis, la vasoconstricción y la toxicidad celular directa, así como la remodelación vascular forman parte de la patofisiología de la NIC. El impacto de los agentes bloqueadores del sistema renina-angiotensina-aldosterona sobre el desarrollo de NIC sigue siendo objeto de debate [19], [20], [21]. Entre los factores de riesgo asociados al desarrollo de NIC, se encuentran la edad avanzada, DM, ICC, HT, disfunción renal, volumen y tipo de MC, exposición a agentes nefrotóxicos, deshidratación y las intervenciones urgentes. Nuestro estudio muestra que la edad avanzada, la DM, los antecedentes de ICC y el volumen total de MC son factores de riesgo independientes de NIC. Aunque el número de pacientes con antecedentes de AC fue significativamente superior en el grupo NIC (+), el análisis de regresión no lo identificó como un factor predictivo independiente. No se observó ningún efecto de los tratamientos con inhibidores de la enzima convertidora de angiotensina/bloqueadores de los receptores de angiotensina (IECA/ARA) sobre la incidencia de NIC.

Una ADE elevada es indicativa de alteraciones en la eritropoyesis, estrés oxidativo elevado, e inflamación crónica. Los datos obtenidos muestran el valor pronóstico de una ADE elevada en diversos contextos cardíacos, como AC, síndrome coronario agudo, ICC, y fibrilación auricular paroxística [9, 22]. La asociación entre una ADE elevada y el desarrollo de NIC ha sido evaluada previamente en pacientes con un diagnóstico de síndrome coronario crónico que se sometieron a una ICP. Los datos muestran un bajo valor predictivo de ADE en el desarrollo de NIC [23]. Por otro lado, ADE ha mostrado tener valor predictivo pare el desarrollo de NIC en pacientes con síndrome coronario agudo (STEMI) [24]. Sin embargo, en nuestro estudio, donde únicamente se incluyó a pacientes con IAMCEST, no observamos ninguna relación entre ADE y el desarrollo de NIC. Existe evidencia de que INL tiene valor pronóstico para eventos mayores en pacientes con infarto de miocardio, siendo además un indicador de inflamación y proteinuria en pacientes con enfermedad renal crónica [25, 26]. INL es un factor de riesgo independiente de NIC, atribuible a la implicación de la inflamación en la patofisiología del NIC. El volumen plaquetario medio mide el tamaño de las plaquetas y su actividad, reflejando el riesgo de carga, morfología, progresión y susceptibilidad a la formación de placas en el vaso sanguíneo. Los resultados obtenidos demuestran que el volumen plaquetario medio es un factor de riesgo independiente de enfermedades cardiovasculares [27]. Las plaquetas de mayor tamaño poseen mayores propiedades trombogénicas. La amplitud de distribución plaquetaria incluye plaquetas de diferentes tamaños, que poseen diferente actividad metabólica y trombogénica en la circulación [28]. Aunque los valores de VPM y ADE fueron significativamente mayores en el grupo NIC(+), únicamente el VPM fue identificado como un factor predictivo independiente de NIC.

### Limitaciones

El presente es un estudio retrospectivo unicéntrico. La evaluación de otros marcadores inflamatorios, como la proteína C-reactiva de alta sensibilidad, podría aportar mayor información sobre el estado inflamatorio, ofreciendo la oportunidad de investigar su posible correlación con los parámetros hemáticos. Los niveles postoperatorios de creatinina se obtuvieron transcurridas al menos 48 horas desde la exposición al contraste. De este modo, podrían haber pasado inadvertidos algunos pacientes que experimentaron un incremento en los niveles de Cr en suero tras el alta.

## Conclusiones

El empleo de medios de contraste está aumentando, a razón de los avances en las técnicas diagnósticas y terapéuticas empleadas en el campo de la cardiología intervencionista. El daño renal inducido por contraste provoca una mayor morbimortalidad, prolonga la estancia hospitalaria, y aumenta los costes. La naturaleza iatrogénica y predecible de la NIC la convierte en una piedra angular para los estudios en curso y en nefrología, debiendo estos centrarse en los factores de riesgo y en las medidas preventivas, diagnósticas y terapéuticas. Una respuesta inflamatoria crónica podría tener un papel fundamental en la patogénesis de la NIC, y los parámetros hematológicos, medidos mediante hemograma ordinario, se postulan como marcadores útiles y prometedores de NIC, cuando se emplean en conjunción con otros factores de riesgo habituales. El VPM y el INL predicen el desarrollo de NIC en pacientes con IAMSEST sometidos a ICP en nuestro estudio. Es necesario realizar más estudios prospectivos destinados a evaluar la relación entre los parámetros hemáticos y la prevalencia de NIC.
